# Application of support vector machine modeling for prediction of common diseases: the case of diabetes and pre-diabetes

**DOI:** 10.1186/1472-6947-10-16

**Published:** 2010-03-22

**Authors:** Wei Yu, Tiebin Liu, Rodolfo Valdez, Marta Gwinn, Muin J Khoury

**Affiliations:** 1 National Office of Public Health Genomics, Coordinating Center for Health Promotion, Centers for Disease Control and Prevention, Atlanta, GA, USA

## Abstract

**Background:**

We present a potentially useful alternative approach based on support vector machine (SVM) techniques to classify persons with and without common diseases. We illustrate the method to detect persons with diabetes and pre-diabetes in a cross-sectional representative sample of the U.S. population.

**Methods:**

We used data from the 1999-2004 National Health and Nutrition Examination Survey (NHANES) to develop and validate SVM models for two classification schemes: Classification Scheme I (diagnosed or undiagnosed diabetes vs. pre-diabetes or no diabetes) and Classification Scheme II (undiagnosed diabetes or pre-diabetes vs. no diabetes). The SVM models were used to select sets of variables that would yield the best classification of individuals into these diabetes categories.

**Results:**

For Classification Scheme I, the set of diabetes-related variables with the best classification performance included family history, age, race and ethnicity, weight, height, waist circumference, body mass index (BMI), and hypertension. For Classification Scheme II, two additional variables--sex and physical activity--were included. The discriminative abilities of the SVM models for Classification Schemes I and II, according to the area under the receiver operating characteristic (ROC) curve, were 83.5% and 73.2%, respectively. The web-based tool-Diabetes Classifier was developed to demonstrate a user-friendly application that allows for individual or group assessment with a configurable, user-defined threshold.

**Conclusions:**

Support vector machine modeling is a promising classification approach for detecting persons with common diseases such as diabetes and pre-diabetes in the population. This approach should be further explored in other complex diseases using common variables.

## Background

A supervised machine learning method, the support vector machine (SVM) algorithm [1], has demonstrated high performance in solving classification problems in many biomedical fields, especially in bioinformatics [2,3]. In contrast to logistic regression, which depends on a pre-determined model to predict the occurrence or not of a binary event by fitting data to a logistic curve, SVM discriminates between two classes by generating a hyperplane that optimally separates classes after the input data have been transformed mathematically into a high-dimensional space. Because the SVM approach is data-driven and model-free, it may have important discriminative power for classification, especially in cases where sample sizes are small and a large number of variables are involved (high-dimensionality space). This technique has recently been used to develop automated classification of diseases and to improve methods for detecting disease in the clinical setting [4,5].

To test the potential power of SVM as an approach for classifying individuals into groups defined by disease status, we chose diabetes as an example. In the U.S., diabetes affects an estimated 23.6 million people, of whom about one third are unaware that they have the disease [6]. Another 57 million people have pre-diabetes, with elevated blood glucose levels that increase their risk of developing diabetes, heart disease, and stroke. Recent studies indicate that diabetes can be prevented by lifestyle changes or pharmacotherapy among persons with pre-diabetes [7-9]. Early screening and diagnosis is thus central to effective prevention strategies [10]. To this end, numerous risk scores and prediction equations have been developed to identify people at high risk of developing diabetes or with pre-diabetes based on common risk factors such as body mass index (BMI) and family history of diabetes [11-13]. For example, a recently published risk calculator uses logistic regression to identify people with pre-diabetes and undiagnosed diabetes by employing combinations of common risk variables [14]. Our objective was to generate an SVM-based approach to distinguish people with either undiagnosed diabetes or pre-diabetes from people without either of these conditions. The variables used to generate the SVM models were limited to simple clinical measurements that do not require laboratory tests. Predictions from this approach were compared with the predictions from logistic regression models containing the same set of variables. A final goal was to demonstrate the applicability of the SVM approach by creating a demonstration web-based classification tool.

## Methods

### Data source

In this study, we used a 1999-2004 data set from the National Health and Nutrition Examination Survey (NHANES) to generate the SVM algorithm. NHANES is an ongoing, cross-sectional, probability sample survey of the U.S. population. It collects demographic, health history, and behavioral information from participants in home interviews. Participants are also invited for detailed physical, physiological, and laboratory examinations that are performed by trained personnel in specially equipped mobile centers [15].

We limited our study to non-pregnant participants aged 20 or older. Participants were considered to have diagnosed diabetes if they answered "yes" to the question "Have you ever been told by a doctor or health professionals that you have diabetes?" Participants who answered "no" to this question but who had a measured fasting plasma glucose ≥ 126 mg/dl were considered to have undiagnosed diabetes; those with a fasting plasma glucose 100-125 mg/dl were considered to have pre-diabetes. Participants with fasting glucose <100 mg/dl were considered to not have diabetes (Table 1).

**Table 1:** Description of the National Health and Nutrition Examination Survey data set used for the study

Diagnostic category	Definition	N	Classification Scheme I	Classification Scheme II
Diagnosed diabetes	Answered "yes" to question "Have you ever been told by a doctor or health professionals that you had diabetes?"	1,266	Cases	Excluded from analysis

Undiagnosed diabetes	Answered "no" to question "Have you ever been told by a doctor or health professionals that you had diabetes?" AND Fasting plasma glucose level ≥ 126 mg/dl	195	Cases	Cases

Pre-diabetes	Fasting plasma glucose level 100-125 mg/dl	1,576	Non-cases	Cases

No diabetes	Fasting plasma glucose level <100 mg/dl	3,277	Non-cases	Non-cases

Notes: Total number of the cases for classification scheme I = 1461

Total number of the non-cases for classification scheme I = 4853

Total number of the cases for classification scheme II = 1709

Total number of the non-cases for classification scheme II = 3206

We devised two different classification schemes (Table 1). In Classification Scheme I, the group of persons with diabetes (diagnosed or undiagnosed) was distinguished from those without diabetes, including persons with pre-diabetes. In Classification Scheme II, the group of persons with either undiagnosed diabetes or pre-diabetes was distinguished from those without diabetes. The models were developed using a sample of 80% of the individuals in each group and validated in the remaining 20%.

### Variable selection

We selected 14 simple variables commonly associated with the risk for diabetes: family history, age, gender, race and ethnicity, weight, height, waist circumference, BMI, hypertension, physical activity, smoking, alcohol use, education, and household income. Variable selection was performed according to an automatic approach developed by Chen et al. [16]. The significance of the automatically selected set of variables was further manually evaluated by fine tuning parameters. The variables included in the final selection were those with the best discriminative performance.

### Model generation

Support Vector Machine (SVM) is a supervised machine learning technique that is widely used in pattern recognition and classification problems. The SVM algorithm performs a classification by constructing a multidimensional hyperplane that optimally discriminates between two classes by maximizing the margin between two data clusters. This algorithm achieves high discriminative power by using special nonlinear functions called kernels to transform the input space into a multidimensional space [17].

**Figure 1:**
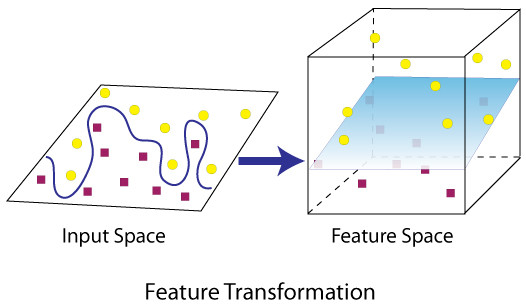
**Demonstration of finding a separating hyperplane in high dimensional space vs in low dimensional space**.

The basic idea behind the SVM technique is to construct an n-1 dimensional separating hyperplane to discriminate two classes in an n-dimensional space. A data point is viewed as an n-dimensional vector. For example, two variables in a dataset will create a two-dimensional space; the separating hyperplane would be a straight line (one dimensional) dividing the space in half. When more dimensions are involved, SVM searches for an optimal separating hyperplane called the maximum-margin separating hyperplane. The distance between the hyperplane and the nearest data point on each side (called support vectors) is maximized. The best scenario is that two classes are separated by a linear hyperplane. However, real-world situations are not always that simple. Some data points in the two classes might fall into a "grey" area that is not easy to be separated. SVM solves this problem by 1) allowing some data points to the wrong side of the hyperplane by introducing a user-specified parameter C that specifies the trade-off between the minimization of the misclassifications and maximization of margin; 2) using kernel functions (usually including linear, polynomial, sigmoid, and radial basis functions (RBF)) to add more dimensions to the low dimensional space, as a result that two classes could be separable in the high dimensional space. Figure 1 shows an example of an inseparable two-dimensional space that becomes separable after the transformation of the input space from low dimensional to multi dimensional. The SVM approach tends to classify entities without providing estimates of the probabilities of class membership in the dataset, which is a fundamental difference from multiple logistic regression.

Two key parameters for the kernels, C and gamma, need to be pre-selected to generate an optimal SVM model. Parameter C controls over-fitting of the model by specifying tolerance for misclassification. Parameter gamma controls the degree of nonlinearity of the model.

We used LibSVM [18], a freely available SVM software library, to generate the SVM models. To generate the data set for model training, we randomly selected a number of non-cases to match the number of cases in the training data set (see Table 1 for the definitions of cases and non-cases). According to the required data format input, values of selected features were normalized to values from - 1 to +1. Values of categorical variables such as Race are arbitrarily assigned to numbers between -1 and +1. For example, -1, -0.5, 0, 0.5, 1 represents non-Hispanic white, non-Hispanic black, Mexican American, other, other Hispanic respectively. Values of continuous variables were transformed into values between -1 and +1 by dividing them by an appropriate number. For example, the age values were divided by 100. In the training data set, the first column of the input data was set to the known outcome, i.e., 1 for positive, - 1 for negative. A utility included in the LibSVM package (grid.py) was used to find the optimal parameters for penalty parameter C and gamma under 5-fold cross-validation. Different kernel functions, including linear, polynomial, sigmoid, and radial basis functions (RBF), were tested and selected for the models based on performance.

Multiple logistic regression modeling (MLR) was performed using the same selected risk variables or features and case status (as specified previously and in Table 1) as the outcome variable. The logistic regression analysis was performed with the training data set using SAS-callable SUDAAN version 9, a procedure specific for complex survey design. Then, the estimated β coefficients were applied to the test data set to calculate for each individual the probability of being a case.

### Model evaluations

#### Evaluation in the test data sets

Test data sets were used to assess the performance of the models. Validation using the test data sets avoided potential bias of the performance estimate due to over-fitting of the model to training data sets. For the SVM model, the data files in the test data sets were formatted according to the requirement that variable values be normalized to values from - 1 to +1; the first column of the input data set (indicating case status) was set to 0. Prediction program Java code from the LibSVM library was modified to output the decision value (internal score generated by SVM tool) for each member of the test data set. For the logistic regression model, the prediction value for each member of the test data set was estimated by using the logistic regression function generated during the training step.

#### 10-fold cross-validation in the training data set

To evaluate the robustness of the estimates from the SVM models, a 10-fold cross-validation was performed in the training data set. The training data set was partitioned into 10 equal-size subsets. Each subset was used as a test data set for a model trained on all cases and an equal number of non-cases randomly selected from the 9 remaining data subsets. This cross-validation process was repeated 10 times, allowing each subset to serve once as the test data set. To generate summary performance estimates, we averaged the area under the curve (AUC) of the receiver operating characteristic (ROC) curve and other statistics (sensitivity, specificity, positive predictive value [PPV], negative predictive value [NPV]) of the cross-validations.

#### Statistics for performance evaluation

ROC curves were generated based on the predicted outcome and true outcome. The AUCs for the test data sets were calculated and used to compare the discriminative powers of the models. We used Delong's method to calculate *P*-values to compare the AUCs based on results of the SVM models and MLR models [19].

Sensitivity, specificity, PPV, and NPV were calculated based on the following formulas when the cutoff value was set to default value (0) in the SVM model.

where TP, FP, TN, and FN represent the number of true positives, false positives, true negatives, and false negatives, respectively.

### Demonstration web-based classification tool implementation

We implemented the SVM model as a web-based tool that we called Diabetes Classifier. The application was built by using J2EE technology [20] and other Java open-source frameworks such as Hibernate [21] and Strut [22]. LibSVM open-source Java codes were modified and embedded in the system source codes for prediction. The lookup tables for cutoff values and corresponding statistics (sensitivity, specificity) were generated from the calculations on each data point in the test data sets. Diabetes Classifier is freely accessible via http://www.hugenavigator.net/DiseaseClassificationPortal/startPageDiabetes.do

## Results and Discussion

**Figure 2:**
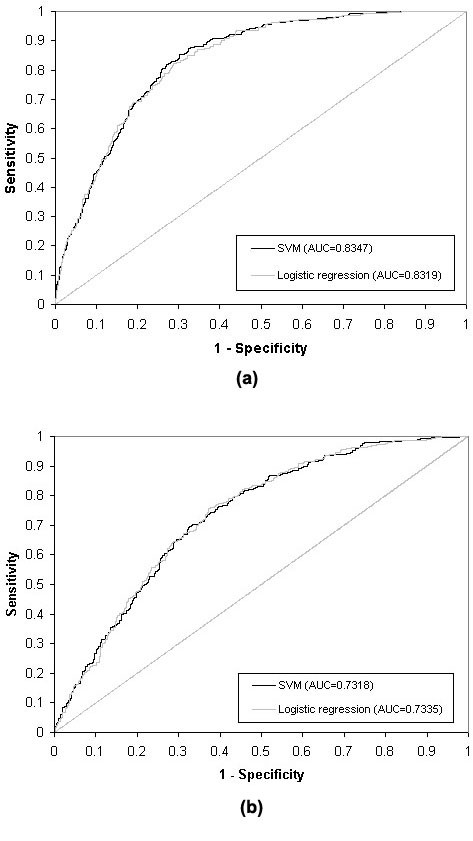
**ROC curves for Classifications Schemes I (a) and II (b) with SVM models and logistic regression models**. Note: see Table 1 for the definitions of Classification Schemes I and II.

In Classification Scheme I (diagnosed or undiagnosed diabetes vs. no diabetes or pre-diabetes), 8 variables--family history, age, race and ethnicity, weight, height, waist circumference, BMI, and hypertension--yielded the best performance. In Classification Scheme II (undiagnosed diabetes or pre-diabetes vs. no diabetes), 10 variables--family history, age, race and ethnicity, weight, height, waist circumference, BMI, hypertension, sex, and physical activity--performed best. Kernel functions were evaluated in terms of their discriminative accuracy by AUC. The RBF kernel function performed best in Classification Scheme I, and the linear kernel function performed best in Classification Scheme II (Table 2). Performance parameters such as the AUC, sensitivity, specificity, positive predictive value, and negative predictive value are presented in Table 3. The overall discriminative ability of Classification Schemes I and II are represented by their AUC values (83.47% and 73.18%, respectively; Figure 2).

**Table 2:** The performance of support vector machine models with four kernel functions for the Classification I and Classification II

Model	Area under the curve			
	
	Linear	Polynomial	Radial basis function	Sigmoid
Classification Scheme I*	0.8332	0.7655	0.8347**	0.8341
Classification Scheme II*	0.7318*	0.6673	0.7259	0.7273

* see Table 1 for the definitions of Classification Schemes I and II

**Best performance

**Table 3:** The performance of support vector machine models for the Classification I and Classification II

Model	Data set	Sensitivity	Specificity	PPV	NPV	AUC
Classification Scheme I*	Test	0.7715	0.7503	0.4926	0.9127	0.8347
	Training	0.7938	0.7169	0.4550	0.9211	0.8383
	10-fold cross- validation	0.7765	0.7027	0.4388	0.9130	0.8242
Classification Scheme II*	Test	0.7359	0.6254	0.5061	0.8195	0.7318
	Training	0.7092	0.6590	0.6729	0.8087	0.7393
	10-fold cross- validation	0.7059	0.6589	0.5293	0.8054	0.7357

PPV, positive predictive value; NPV, negative predictive value; AUC, area under the curve.

*See Table 1 for the definitions of Classification Schemes I and II.

The AUC values for logistic regression analyses of the Classification Schemes I and II were 83.19% and 73.35%, respectively (Figure 2). Comparing the AUCs from our SVM and MLR models revealed no statistically significant difference in their discriminative abilities (*P *= 0.3672 and *P *= 0.6718 for Classification Schemes I and II, respectively); thus, the SVM approach appears to perform as well as the traditional logistic regression model.

Diabetes Classifier, the web-based demonstration tool, was built based on the two SVM models. By selecting one of the diabetes classifications, the user is asked to enter the values for 8 or 10 common variables; the classification result is then presented on the next page, using the default cutoff value (0). This application provides an interface that allows the user to select the cutoff values. Each cutoff value has specific values of sensitivity and specificity so that the user can decide how the tool could be used in screening for diabetes. Diabetes Classifier can also be used in batch mode to classify observations in an uploaded file containing appropriately formatted values of required variables.

## Conclusions

In this study, we tested two classification schemes to detect cases of diabetes and pre-diabetes in the U.S. population. Both schemes are examples of the potential use of support vector machine techniques in the classification of common diseases. Our results demonstrated that the discriminative performance of SVM models was equivalent to the epidemiological method commonly used for this purpose, multivariate logistic regression. To our knowledge, this is the first report that the SVM approach can be used successfully to detect a common disease with simple clinical measurements, without laboratory tests. Based on these results, we also developed a web-based tool for classification of diabetes and pre-diabetes. This tool demonstrates useful features for the potential application of classification algorithms in health care.

SVM is a model-free method that provides efficient solutions to classification problems without any assumption regarding the distribution and interdependency of the data. In epidemiologic studies and population health surveys, the SVM technique has the potential to perform better than traditional statistical methods like logistic regression, especially in situations that include multivariate risk factors with small effects (e.g., genome-wide association data and gene expression profiles), limited sample size, and a limited knowledge of underlying biological relationships among risk factors. This is particularly true in the case of common complex diseases where many risk factors, including gene-gene interactions and gene-environment interactions, have to be considered to reach sufficient discriminative power in prediction models [23]. Our work provides a promising proof of principle by demonstrating the predictive power of the SVM with just a small set of variables. This approach can be extended to include large data sets, including many other variables, such as genetic biomarkers, as data become available.

A major strength of this study is that we used the NHANES data set, which is a unique national weighted survey data that is representative of the U.S. population. Our results are comparable to those of other models tested in the same population. For example, Keikes et al. [24] developed a tool for detecting undiagnosed diabetes and pre-diabetes using logistic regression and a classification tree method to predict the risk of the diabetes in the U.S. population. Although direct comparisons are difficult because of the use of different NHANES data sets and different validation strategies, the discriminative powers in both studies seem to be equivalent. In our study, the AUC for the detection of diagnosed diabetes or undiagnosed diabetes was 83.47%, and it was 73.18% for pre-diabetes or undiagnosed diabetes in the validation test. In the study from Keikes et al., the AUC for undiagnosed diabetes were 82.19% (5-fold cross-validation) and 75.03% (training data set) for pre-diabetes or undiagnosed diabetes. Schwarz et al. [25] recently published a comprehensive review of existing tools for predicting the risk of type 2 diabetes or detecting undiagnosed diabetes. These tools were developed for different populations under different methodologies using different sets of variables. In general, the discriminative power of our SVM method is within the range of discriminative powers reported for the tools included in this review.

We cannot be certain that the models we developed by using the particular NHANES data set described here are applicable to other populations. Our SVM approach, however, is easily extended to other populations to generate their own classification systems. Likewise, a similar approach could be used to develop SVM models for other complex diseases using a different set of relevant variables.

A critical step for determining the usefulness of a screening test is to establish optimal cutoff values that yield optimal sensitivity and specificity values, which are particularly important for cost-effectiveness analysis [26]. Our web-based application, Diabetes Classifier, displays the trade-offs in sensitivity and specificity of the classification method as the cutoff value is changed. This feature is particularly relevant to clinical and public health programs, which can configure cutoff scores according to the objectives of the program and other considerations including cost-effectiveness. Diabetes Classifier allows data to be fed automatically (via data batch file uploading) for classification and provides an interface capable of sharing information with other sectors of a health care system. Web-based tools such as Diabetes Classifier can also serve as self-assessment tools for use by the general public.

Support vector machine modeling is a promising classification approach for detecting a complex disease like diabetes using common, simple variables. Validation indicated that the discriminative powers of our two SVM models are comparable to those of commonly used multivariable logistic regression methods. Our Diabetes Classifier tool, a web-based tool developed for demonstration purposes only, illustrates a potential use of the SVM technique: the identification of people with undetected common diseases such as diabetes and pre-diabetes. This approach needs to tested and validated in other studies.

## Competing interests

The authors declare that they have no competing interests.

## Authors' contributions

WY designed and developed the methodology, built the demo web-based system, and drafted the manuscript. TL performed the data preparation and statistical analysis, RV provided expertise on diabetes and helped in manuscript preparation. MG provided advice on the project and revised the draft manuscript. MJK oversaw the project and revised the draft manuscript. All authors read and approved the final document.

## Pre-publication history

The pre-publication history for this paper can be accessed here:

http://www.biomedcentral.com/1472-6947/10/16/prepub
